# Maternal Employment and Child Survival During the Era of Sustainable Development Goals: Insights from Proportional Hazards Modelling of Nigeria Birth History Data

**DOI:** 10.29024/aogh.11

**Published:** 2018-04-30

**Authors:** Joshua O. Akinyemi, Bola L. Solanke, Clifford O. Odimegwu

**Affiliations:** 1 Demography and Population Studies Programme, Schools of Public Health and Social Sciences, University of the Witwatersrand, Johannesburg, ZA; 2 Department of Epidemiology and Medical Statistics, Faculty of Public Health, College of Medicine, University of Ibadan, Ibadan, NG; 3 Department of Demography and Social Statistics, Obafemi Awolowo University, Ile Ife, NG

## Abstract

**Background:**

One of the targets for the third and fifth Sustainable Development Goals (SDGs) borders on children survival and women economic empowerment, respectively. A robust investigation of the relationship between maternal employment and childhood mortality will provide information useful for programs aimed at ensuring the complementarity of SDG 3 (healthy life for all) and SDG 5 (gender equality, girls and women empowerment).

**Objective:**

We addressed the following questions: (1) What is the independent relationship between maternal employment and infant (0–11 months) and child (12–59 months) mortality in Nigeria? (2) How does father’s occupation, type of residence, and geopolitical region modify the relationship?

**Methods:**

We retrospectively analysed cross-sectional data on weighted sample of 31,828 under-five children extracted from the birth history in the 2013 round of Nigeria Demographic and Health Survey, using Cox proportional hazards models. The outcomes of interest were infant (0–11 months) and child (12–59 months) mortality, and the main explanatory variables include maternal employment, involvement in decision making on work earnings, and father’s occupation. Other confounding variables were also controlled.

**Findings:**

Results showed that about two-third (68.7%) of under-five children had mothers who were working, with the majority engaged in self-employed occupations such as sales or small businesses, agriculture, and other manual labour. Infant mortality rate amongst children of employed mothers (65 per 1000 live births) was slightly less than the unemployed (70 per 1000 live births). A similar pattern was observed for child mortality. Hazards regression models revealed that the risk of both infant and child mortality was higher amongst unemployed women. Sales and agriculture/manual occupation constituted a higher risk for infant and child mortality. Analysis of interaction effects also revealed variations by father’s occupation, type of residence, and geopolitical region.

**Conclusion:**

The role of maternal employment in child survival is dynamic and depends on the type of occupation, family, and residential and regional context.

## Background

The pursuit of Sustainable Development Goal (SDG) has commenced and will be on for the next 14 years. The 17 goals, which are all inter-related and cross-cutting, touched on several critical aspects of health and development. This study pertains to the interconnection between the third (SDG 3) and fifth (SDG 5) goal, which focused on healthy life at all ages and gender equality and empowerment for all girls and women, respectively [1]. The second target under SDG 3 aimed at reducing under-five mortality to less than 25 per 1000 live births by year 2030 [2]. At least two different targets under SDG 5 aimed to place economic resources in the hands of women. This requires increased participation of women in the labour force, which was also a target in the forerunner third millennium development goal. The world development report indicated that female employment in sub-Saharan Africa increased from 57.1% in 1990 to 63.0% in 2014 [3]. Under-five mortality declined by 52% between 1990 (179 per 1000 live births) and 2015 (86 per 1000 live births) over the same period [4]. Women employment enhances household income and translates into better standard of living especially for children [5]. The literature also suggests that women employment affect demographic outcomes such as fertility and childhood mortality [6].

Effects of women employment on fertility is relatively better documented in the literature [6,7,8]. Though evidence on the relationship between women employment and fertility could be argued to be mixed depending on the study setting, the predominant finding is that female labour force participation is associated with lower fertility [7]. The relationship is often attributed to the role conflict between employment and childbearing responsibilities [6]. A woman who is employed, especially outside the home, would most likely want to avoid large family size so that she can cope with the demands and responsibilities at the workplace.

Concerning childhood mortality, there is no convincing evidence on the role of maternal employment, especially in developing countries. Some studies suggest that children of women who are employed have higher risk of mortality because such women do not have adequate time for child care [9,10]. It is noted that most of the available literature on this relationship date back to the 1990s and therefore may not reflect the current realities. In contrast, other evidence from developed setting suggests that maternal employment reduced the risk of small birth size and infant mortality [11]. A comprehensive investigation based on data from nine countries in south and south-east Asia also showed mixed findings [12]. For instance, while there was a positive relationship between maternal employment and birth size in Nepal, a negative association was observed in India [12]. Another feature in many of these studies was that once socio-economic status (proxied by education and household wealth status) was adjusted, the significant effect of maternal employment often disappeared [13]. It thus appears that maternal employment is not important for child health and survival. This pattern of results in previous studies could be attributed to the manner in which the variable, maternal employment, was coded in many of the analyses. Most often, a dichotomous classification – employed versus not employed – was used. This classification masked very important variations that could be introduced due to the type of occupation in which a woman is engaged. It should be expected that there would be difference in the child health outcomes for women employed in the formal sector (private or public) and those in agriculture and other forms of self-employment, which expose children to adverse environmental hazards [14,15,16].

The mechanism behind the relationship between maternal employment and child survival is similar to that of fertility. Inability of working mothers to devote time to child care is a plausible explanation [7,8]. If a woman is employed in a job that is compatible with childcare responsibilities, then the relationship would not be negative. This expectation was confirmed by Ukwuani et al. [17], who found that nutritional status was better amongst children whose mothers work outside the home and were able to take them along. For women who work in the formal sector, where there is opportunity for maternity leave, employment may not have a negative effect on children survival, especially in the neonatal period. Unfortunately, the majority of women in sub-Saharan Africa work in the informal sector, where such opportunities are not feasible [3].

The current drive to promote female labour force participation and reduce childhood morbidity and mortality could be potentially hampered without proper balancing. This is because the type of occupation in which women are employed have both direct and indirect consequence for child care [15]. Updated evidence on the relationship between maternal employment and childhood mortality are therefore needed to make suggestions useful to fine tune policies and programmes in pursuit of SDG 3 and SDG 5. This is sacrosanct, especially in sub-Saharan Africa (SSA), where the magnitude of childhood mortality and slow progress in its reduction is a perennial concern. Therefore, this study is aimed at reappraising the relationship between maternal employment and childhood mortality in SSA. Nigeria, the most populous country has one of the highest childhood mortality rates in the region, was used as a case study.

## The Nigeria Context

Although the female labour force participation in Nigeria increased from 39% in 1990 to 48.3% in 2015, the percentage of women employed and paid cash in non-agricultural occupation rose from 35% to 41.0% over the same period [3]. The most common occupation among women aged 15–49 years are sales, services (60.5%), and agriculture (15.6%) [18]. Two-thirds of the population reside in rural areas, where access to modern employment and infrastructure is limited [18]. Under-five mortality levels and trends between 1990 and 2015 differ across the six geopolitical regions, with northern regions having higher magnitude [19,20]. Regional differences in childhood mortality have been linked to variations in the socio-economic characteristics and utilisation of maternal and child health care services [21,22,23]. Studies that focussed on the relationship between maternal employment and childhood mortality are rare, though a few included maternal employment as a control variable [24,25,26].

A community-based study conducted in the 1990s in south-western Nigeria reported that maternal employment was associated with higher risk of infant mortality, which was even higher in children whose mothers work in agricultural and other informal sector [9]. The author argued that this negative relationship could be due to non-availability of adequate time for childcare amongst working mothers. Some other studies merely controlled for maternal occupation while investigating factors associated with childhood immunization [27] or under-five mortality [24]. In these studies, maternal occupation was categorised as: (1) professional/technical/managerial, (2) clerical/sales/services/skilled manual, (3) agricultural/unskilled/domestic, and (4) not working. The two studies did not found any difference in the mortality risks across the different occupational groupings. However, two limitations are notable. First, they were based on the 2003 Nigeria Demographic and Health Survey, which has the smallest sample size for children amongst the five rounds of DHS in Nigeria, thereby limiting the statistical power to detect any difference. Second, the categories used for maternal occupation were not very discriminatory for childhood mortality. This is especially applicable to the second category, clerical/sales/services/skilled manual. This category included women in the formal (clerical and services) and informal (sales/skilled manual) sectors, both of which could affect child health outcomes in different ways.

Another study which focused on intimate partner violence and under-five mortality in Nigeria controlled for maternal occupation and categorised it as none, skilled, and unskilled [25]. The results showed that there was no association between maternal occupation and under-five mortality. Again, the classification used for maternal occupation suffered similar limitation, as described earlier. The skilled group could be found in both formal and informal sectors, and they could be employees or self-employed.

Furthermore, a study which aimed to compare the correlates of infant mortality between north-east and south-west Nigeria also included maternal occupation as a dichotomous variable (working and not working). Findings showed that maternal work was associated with lower infant mortality in the south-west, but the reverse was the case in the north-east [26]. Though the authors offered no possible explanation for this variation, differences in occupation type between the two regions may be responsible for this. Also, the binary classification used in the study was too broad and limited in providing a clear understanding of the relationship between maternal employment and childhood mortality. Several other studies on childhood mortality in Nigeria did not control for maternal or paternal employment [19,28,29].

In summary, previous studies about the relationship between maternal employment and childhood mortality in Nigeria depict some knowledge gaps, which the present study was designed to fill. First, the only known study which focussed on the subject matter [9] is quite old because it was based on data collected in 1980–81. Besides, it could neither represent the south-west region nor the entire country. Second, the few studies that controlled for maternal occupation did not use a uniform classification, and the findings could not enhance a deeper understanding of the relationship between maternal employment and child survival. Third, apart from maternal occupation, no other employment-related variables were included in the analyses. Partner’s occupation could enrich the knowledge about the subject. A robust investigation of the relationship between maternal employment and childhood mortality will provide information useful for programs aimed at ensuring the complementarity of SDG 3 (healthy life for all) and SDG 5 (gender equality, girls and women empowerment). In view of these, the present study addressed the following questions: (1) What is the independent relationship between maternal employment and infant (0–11 months) and child (12–59 months) mortality in Nigeria? (2) How does father’s occupation, residence, and geopolitical region modify the relationship?

## Methods

### Data

A weighted sample of 31,828 in the children recode data file from the 2013 round of Nigeria Demographic and Health Survey (NDHS) was utilised for this study. The file was created from data collected on women background characteristics and reproductive history. In brief, the nationally representative NDHS 2013 involved the use of a two-stage cluster sampling technique to select eligible women aged 15–49 years. The survey was designed to provide estimates at national and regional levels, as well as for the 36 states in Nigeria. Enumeration areas developed for the 2006 population and housing census were used as primary sampling units. Detailed information about the questionnaires and sampling design of the NDHS 2013 are available in the published report [30].

### Analytical Framework and Study Variables

**Figure 1:**
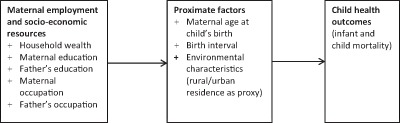
Analytical framework (Adapted from Rodgers 2011, 48).

This study is premised on the “proximate determinants framework” as expatiated by Rodgers [12]. Maternal employment was assumed to enhance household’s socio-economic resources, which in turn operate through a set of intermediate or proximate variables to influence child health outcomes such as mortality (Figure 1).

Two outcome measures were used in this study. These were infant (0–11 months) and child (12–59 months) mortality, which were analysed as event history outcomes. As such, the survival time was age at death (in months). Children who survived the period 0–59 months were censored at their current age at the time of survey data collection.

The main explanatory variable was maternal occupation, which captured the actual work engaged in by mothers of under-five children, and it was divided into four categories: (1) not working, (2) professional/services, (3) sales/trading, and (4) agriculture/manual labour. Another variable provided indication on whether the mother of an under-five is involved in decision making on how earnings from her work is spent. It also has three categories: (1) involved, (2) not involved, and (3) not working or not earning cash from work. The last employment variable was father’s occupation, which has the same categories as maternal occupation.

Based on evidence from previous studies, several other variables were controlled during the analyses. The control variables are maternal education, father’s education, and household wealth quintile. These variables are indicators of household socio-economic status and are known to be negatively related to childhood mortality. For example, children of mothers with secondary or higher education, and those living in rich households, have lower risks of death [31,32]. Other variables include maternal age at child’s birth, preceding birth interval, marital status, residence, and geo-political region [28,33].

### Analysis

The first stage in the analysis was a description of the employment profile and other variables using frequencies and percentages. At the second stage, the synthetic cohort life table technique was employed to estimate infant and child mortality rates disaggregated by employment variables. The mortality rates were compared using the log rank test. Last, Cox proportional hazards models were fitted to investigate the relationship between maternal employment and childhood mortality. Four sets of hierarchical models were fitted as exemplified by the conceptual model for child health outcomes [34]. The first set were univariate models (model 0), in which the independent variables were included one at a time. Second (model 1), all employment variables were entered into the model to ascertain the relative importance of each of them. In the third (model 2), maternal education, father’s education, and household wealth quintile was added to model 1. The full model (model 3) included all variables so that the independent relationship with childhood mortality can be assessed. Separate models were fitted for infant (0–11 months) and child (12–59 months) mortality. To get a deeper understanding of the different context of maternal employment, interaction effects with residence, father’s occupation, and region was investigated. In the multivariate analyses, measure of effect was estimated as hazard ratio (HR) with 95% confidence interval (CI), while the Wald test was used to assess the statistical significance of the model. Multicollinearity was assessed using variance inflation factor [35].

Data was weighted to account for the complex sample design of the NDHS 2013. Besides, for the hazards regression, robust standard errors were estimated to control for clustering of observations from the same primary sampling unit. Stata SE version 12.0 was used for all analyses.

### Ethical Issues

This study was a secondary analysis of data from NDHS 2013. The survey got ethical approval from the National Health Research Ethics Committee in Nigeria with approval number NHREC/2008/07. Prior to data collection, informed consent was obtained from all participants. Finally, necessary approval was obtained to download the data from DHS archive.

## Results

### Maternal Employment and Background Characteristics

The employment profile of mothers and fathers of under-five children in the NDHS 2013 are summarised in Table 1. About two-thirds (68.7%) of under-five children had mothers who were working at the time of survey. The majority were engaged in self-employed occupations such as sales or small businesses and agricultural and other manual labour. The results showed that most of them (56.2%) were involved in decision making on how their cash earnings are spent. Virtually all children had working fathers, with the majority engaged in agriculture and manual labour (60.1%).

**Table 1:** Employment profile amongst parents of under-five children in Nigeria, NDHS, 2013.

Variables	No. of Children	%

**Mother Currently Employed**		
Yes	21,865	68.7
No	9,964	31.3
**Type of Employment**		
employed by others	3,231	10.2
self employed	19,097	60.0
not employed	9,500	29.8
**Maternal Occupation**		
not working	9,437	29.7
professional/services	2,631	8.3
Sales	12,514	39.3
agriculture/manual	7,247	22.8
**Involvement in Decision on Earnings**		
involved	17,898	56.2
not involved	2,030	6.4
not working/no cash earning	11,899	37.4
**Father’s Occupation**		
not working	926	2.9
professional/services	5,510	17.3
Sales	6,258	19.7
agriculture/manual	19,134	60.1

Table 2 presents the demographic characteristics according to type of maternal employment. Overall, 70.4% of under-five children were born to women aged 20–35 years. Almost half (49.2%) had mothers with no formal education, while 31.5% attained at least secondary education. Distribution of paternal education showed that 40.2% had fathers with secondary or higher education. Though 34.4% lived in households within the rich wealth quintile, 65.0% were domiciled in rural areas. The north-west region (37.0%) and south-east (8.9%) contributed the largest and smallest proportion of children, respectively, to the sample.

**Table 2:** Background characteristics according to type of maternal employment.

Variables	Total Sample		Maternal Occupation			Agriculture/manual

	N	%	Not working	Professional/services	Sales	

<20 yrs	4,002	12.6	49.7	3.9	26.6	19.8
20–35 yrs	22,394	70.4	28.6	9.2	39.6	22.6
above 35 yrs	5,432	17.0	19.4	7.6	47.4	25.6
**Maternal Education**						
no formal education	15,657	49.2	37.2	2.0	39.8	21.1
primary	6,127	19.3	19.4	5.6	39.8	35.2
secondary/higher	10,044	31.5	24.1	19.8	38.3	17.8
**Paternal Education**						
no formal education	13,142	41.3	39.2	2.6	38.5	19.8
primary	5,884	18.5	21.5	6.1	40.4	32
secondary/higher	12,802	40.2	23.6	15.1	39.7	21.6
**Marital Status**						
not currently married	1,337	4.2	31.7	11.2	34.7	22.4
currently married	30,491	95.8	29.6	8.1	39.5	22.8
**Household Wealth Index**						
Poor	14,851	46.7	35.2	2.2	36.5	26.1
Middle	6,001	18.9	28.5	6.0	37.5	28
Rich	10,976	34.4	22.8	17.8	44.1	15.4
**Residence**						
Rural	20,702	65.0	32.4	4.6	37.3	25.6
Urban	11,126	35.0	24.5	15.0	43.1	17.4
**Region**						
North-Central	4,340	13.6	20.2	7.5	37.5	34.9
North-East	5,578	17.5	48.9	3.6	23.5	24
North-West	11,775	37.0	34.4	3.0	45.3	17
South-East	2,840	8.9	21.6	16.2	37.2	25.1
South-South	2,935	9.2	22.5	16.4	36.0	25.1
South-West	4,360	13.7	11.9	18.4	48.9	20.8
**Birth interval**						
first birth	6,285	19.8	41.1	12.5	27.8	18.7
≤24 months	6,813	21.4	30.5	7.2	38.6	23.7
25–36 months	9,677	30.4	27.3	7.0	42.0	23.8
above 36 months	9,052	28.4	23.6	7.6	45.1	23.8

Further details from Table 2 showed that the percentage of children whose mothers were not employed decreased by mother’s age group. Amongst children whose mothers had no formal education, the greatest proportion were unemployed (37.2%), while the percentage employed in the professional and services sector (19.8%) was highest amongst children whose mothers had secondary or higher education. The proportion of children with unemployed mothers was higher in the rural than urban areas. Regional distribution of maternal occupation revealed that the north-east (48.39%) and north-west (34.4%) had the greatest number of children whose mothers were not employed. The south-west recorded the least (12.0%).

### Maternal Employment and Infant Mortality

Table 3 presents the infant (0–11 months) and child (12–59 months) mortality rates disaggregated by different measures of maternal employment. The infant mortality rate amongst children of employed mothers (65 per 1,000 live births) was slightly less than those not employed (70 per 1,000 live births). Children whose fathers were engaged in agriculture and manual labour had the highest infant mortality. The log rank tests also showed that there was significant difference in the infant mortality rates across father’s occupation.

**Table 3:** Infant and child mortality rates disaggregated by maternal employment profile in Nigeria, NDHS, 2013.

Variables	Mortality Rates (per 1000 live births)					
	0–11 Months			12–59 Months		

	IMR	Log-Rank Test; p-value		CMR	Log-Rank Test; p-value	

**Maternal Occupation**						
not working	70			50		
professional/services	59			15		
sales	64			52		
agriculture/manual	69	4.06; 0.2556		47	25.3; <0.001	*
**Type of Employment**						
employed by others	64			39		
self employed	65			47		
not employed	71	2.73; 0.2556		50	3.45; 0.1786	
**Involvement in Decision on Earnings**						
involved	64			48		
not involved	57			38		
not working	72	7.12; 0.0285	*	48	1.28; 0.5266	
**Father’s Occupation**						
not working	62			24		
professional/services	54			36		
Sales	65			51		
agriculture/manual	71	11.68; 0.0086	*	50	11.92; 0.0077	*

IMR = infant mortality rate; CMR = child mortality rate. * p < 0.05.

Results from multivariate Cox hazards regression models fitted to explore the relationship between maternal employment and infant mortality are summarised in Table 4. In the univariate models (model 0), though not statistically significant, the risk of infant death was higher in children of women who were not employed (HR = 1.18, CI: 0.89–1.55) or those engaged in agriculture and manual work (HR = 1.16, CI: 0.87–1.54), compared to women in professional and services occupation. Paternal occupation, maternal age at child’s birth, maternal education, paternal education, household wealth index, residence, and birth interval were also significantly associated with infant death. Model 1 showed that the types of fathers’ occupations were significantly related to infant death. Children whose fathers’ occupation was agriculture and manual labour were 1.27 times as likely to die during infancy (HR = 1.27, CI: 1.10–1.47), relative to those whose fathers were professionals. Controlling for maternal and paternal education as well as household wealth index (Model 2), children whose mothers were not working were less likely to suffer infant death (HR = 0.78, CI: 0.56–1.09). This pattern was also repeated in the full model, in which the hazard of infant death was lowest amongst children of women not employed (HR = 0.83, CI: 0.59–1.15). The full model also showed that middle and rich wealth quintile, as well as urban residence, was associated with lower hazards of infant death.

**Table 4:** Relationship between maternal employment and infant mortality adjusted for other variables, Nigeria, DHS, 2013.

Variables	Model 0		Model 1		Model 2		Model 3	

	HR (95% CI)		HR (95% CI)		HR (95% CI)		HR (95% CI)	

**Maternal Occupation**								
not working	1.18 (0.89–1.55)		0.95 (0.67–1.34)		0.78 (0.56–1.09)		0.83 (0.59–1.15)	
professional/services	1.00		1.00		1.00		1.00	
sales	1.07 (0.81–1.41)		1.03 (0.76–1.35)		0.88 (0.66–1.17)		0.90 (0.67–1.20)	
agriculture/manual	1.16 (0.87–1.54)		1.07 (0.80–1.42)		0.89 (0.65–1.20)		0.91 (0.67–1.25)	
**Involvement in Decision on Earnings**								
involved	1		1.0		1.0		1	
not involved	0.89 (0.68–1.18)		0.88 (0.68–1.14)		0.89 (0.69–1.15)		0.89 (0.68–1.15)	
not working/earning cash	1.12 (0.98–1.29)		1.20 (0.98–1.48)		1.21 (0.99–1.47)		1.10 (0.87–1.40)	
**Father’s Occupation**								
not working	1.15 (0.83–1.58)		1.07 (0.76–1.49)		1.05 (0.73–1.52)		0.89 (0.62–1.29)	
professional/services	1.00		1.00		1.00		1.00	
Sales	1.19 (0.93–1.53)		1.18 (0.93–1.51)		1.06 (0.86–1.32)		1.04 (0.83–1.30)	
agriculture/manual	1.29 (1.11–1.49)	*	1.27 (1.10–1.47)	*	1.09 (0.95–1.25)		1.10 (0.95–1.28)	
**Maternal Age at Child’s Birth**								
<0 yrs	1						1	
20–35 yrs	0.73 (0.63–0.85)	*					0.91 (0.77–1.08)	
above 35 yrs	0.87 (0.68–1.12)						1.05 (0.77–1.42)	
**Maternal Education**								
no formal education	1				1.0		1	
Primary	0.93 (0.76–1.14)				1.06 (0.90–1.24)		1.03 (0.86–1.24)	
secondary/higher	0.68 (0.56–0.84)	*			0.85 (0.67–1.06)		0.79 (0.60–1.02)	
**Paternal Education**								
no formal education	1				1.0		1	
Primary	0.87 (0.73–1.05)				0.96 (0.81–1.13)		0.98 (0.83–1.14)	
secondary/higher	0.73 (0.62–0.87)	*			0.96 (0.79–1.15)		1.01 (0.84–1.21)	
**Marital Status**								
currently married	0.79 (0.62–1.00)						0.77 (0.55–1.08)	
not currently married	1.00						1.00	
**Household Wealth Index**								
poor	1				1.0		1	
middle	0.75 (0.62–0.89)	*			0.78 (0.66–0.93)	*	0.83 (0.71–0.97)	*
Rich	0.66 (0.54–0.79)	*			0.75 (0.61–0.93)	*	0.88 (0.69–1.11)	
**Residence**								
rural	1.00						1.00	
urban	0.72 (0.60–0.87)	*					0.82 (0.69–0.96)	*
**Region**								
North-Central	1.10 (0.89–1.36)						0.90 (0.71–1.15)	
North-East	1.24 (0.91–1.69)						0.88 (0.64–1.22)	
North-West	1.41 (1.09–1.83)	*					1.03 (0.77–1.36)	
South-East	1.41 (1.11–1.79)	*					1.28 (1.02–1.61)	*
South-South	0.97 (0.78–1.20)						0.85 (0.68–1.07)	
South-West	1						1	
**Birth Interval**								
first birth	1.79 (1.47–2.17)	*					2.04 (1.64–2.54)	*
≤ 24 months	2.15 (1.79–2.58)	*					2.21 (1.85–2.64)	*
25–36 months	1.35 (1.15–1.59)	*					1.37 (1.17–1.59)	*
above 36 months	1						1	

* p < 0.05.

### Maternal Employment and Child Mortality (12–59 Months)

Estimates of child mortality rate was lowest for children of women employed in professional and services (formal) sectors (15 per 1,000 children surviving to 1 year) and highest amongst those whose mothers were not working and in those employed in agriculture and manual labour (Table 3). A similar pattern was observed when the rates were disaggregated by paternal occupation.

Results from univariate models (Table 5) showed a pattern different from that of infant mortality. Model 0 revealed that the risk of death (12–59 months) was higher in children whose mothers were not working (HR = 2.92, CI: 1.85–4.59), employed in sales (HR = 3.09, CI: 1.98–4.82), and agriculture and manual (HR = 2.72, CI: 1.69–4.38), compared to those in professional and services employment. All the other variables in the univariate model maintained their usual relationship with child mortality.

**Table 5:** Relationship between maternal employment and child mortality adjusted for other variables, Nigeria, DHS, 2013.

Variables	Model 0		Model 1		Model 2		Model 3	

	HR (95% CI)		HR (95% CI)		HR (95% CI)		HR (95% CI)	

**Maternal Occupation**								
not working	2.92 (1.85–4.59)	*	2.89 (1.63–5.11)	*	1.51 (0.90–2.51)		1.49 (0.89–2.48)	
professional/services	1.00		1.00		1.00		1.00	
sales	3.09 (1.98–4.82)	*	2.91 (1.86–4.57)	*	1.77 (1.18–2.66)	*	1.80 (1.20–2.68)	*
agriculture/manual	2.72 (1.69–4.38)	*	2.57 (1.58–4.20)	*	1.43 (0.89–2.29)		1.48 (0.93–2.36)	
**Involvement in Decision on Earnings**								
involved	1.00		1.00		1.00		1.00	
not involved	0.82 (0.59–1.12)		0.81 (0.59–1.11)		0.84 (0.62–1.14)		0.91 (0.65–1.27)	
not working/not earning cash	1.00 (0.82–1.23)		0.94 (0.69–1.27)		0.93 (0.68–1.27)		0.89 (0.65–1.22)	
**Father’s Occupation**								
not working	0.63 (0.34–1.14)		0.63 (0.34–1.15)		0.62 (0.36–1.09)		0.58 (0.32–1.06)	
professional/services	1.00		1.00		1.00		1.00	
sales	1.30 (0.98–1.73)		1.20 (0.90–1.60)		0.85 (0.63–1.13)		0.82 (0.62–1.09)	
agriculture/manual	1.36 (1.06–1.74)	*	1.26 (0.98–1.62)		0.79 (0.63–0.99)	*	0.81 (0.65–1.02)	
**Maternal Age at Child’s Birth**								
<20 yrs	1.00						1.00	
20–35 yrs	0.60 (0.47–0.77)	*					0.64 (0.50–0.82)	*
above 35 yrs	0.68 (0.54–0.85)	*					0.62 (0.45–0.86)	*
**Maternal Education**								
no formal education	1.00				1.00		1.00	
primary	0.65 (0.51–0.83)	*			0.87 (0.68–1.10)		0.92 (0.71–1.18)	
secondary/higher	0.30 (0.22–0.42)	*			0.61 (0.41–0.91)	*	0.66 (0.44–0.99)	*
**Paternal Education**								
no formal education	1.00				1.00		1.00	
primary	0.83 (0.66–1.03)				1.15 (0.95–1.39)		1.17 (0.98–1.40)	
secondary/higher	0.42 (0.32–0.55)	*			0.91 (0.69–1.20)		0.95 (0.73–1.24)	
**Marital status**								
currently married	1.24 (0.80–1.91)						0.84 (0.53–1.35)	
not currently married	1.00						1.00	
**Household Wealth Index**								
poor	1.00				1.00		1.00	
middle	0.50 (0.39–0.63)	*			0.56 (0.45–0.69)	*	0.65 (0.53–0.82)	*
rich	0.28 (0.21–0.37)	*			0.37 (0.28–0.50)	*	0.53 (0.39–0.72)	*
**Residence**								
rural	1.00						1.00	
urban	0.40 (0.29–0.55)	*					0.70 (0.55–0.89)	*
**Region**								
North-Central	1.44 (0.88–2.37)						0.84 (0.55–1.29)	
North-East	2.85 (1.64–4.95)	*					1.33 (0.83–2.14)	
North-West	3.19 (1.94–5.29)	*					1.33 (0.86–2.04)	
South-East	1.59 (0.89–2.84)						1.48 (0.92–2.39)	
South-South	1.36 (0.84–2.21)						1.19 (0.79–1.80)	
South-West	1.00						1.00	
**Birth Interval**								
first birth	1.40 (1.02–1.920	*					1.44 (1.05–1.97)	*
≤24 months	2.17 (1.81–2.61)	*					2.02 (1.66–2.45)	*
25–36 months	1.57 (1.21–2.02)	*					1.46 (1.11–1.91)	*
above 36 months	1.00						1.00	

* p < 0.05.

Hazard ratios from model 1, which controlled for other employment-related variables, showed that the type of maternal occupation retained its statistical significance such that children of unemployed women and those engaged in sales (HR = 2.89, CI:1.63–5.11) and agriculture and manual labour (HR = 2.57, CI: 1.58–4.20) were more likely to die between the 12th and 59th month of life. Furthermore, when education and household wealth quintile were controlled (Model 2), sales and agricultural occupation remained associated with higher risks of child mortality. From the full model, it was found that the hazards of child death were significantly greater amongst women employed in sales (HR = 1.80, CI: 1.20–2.68). The risk of child death was also higher in those engaged in agriculture but was not statistically significant. Other variables which were associated with lower risk of child death include maternal age at child’s birth (20–35 years), secondary or higher education, middle and rich household wealth quintile, and urban residence. Another factor found to increase the risk was birth interval less than 24 months (HR = 2.02, CI: 1.66–2.45).

### Interaction Effects

#### Infant Mortality (0–11 Months)

Table 6a shows the interaction effects of maternal occupation, type of residence, and father’s occupation on infant mortality. In the urban areas, infants of women who were not employed (HR = 0.63, CI: 0.36–1.11) were less likely to die compared to those with mothers engaged in professional and services occupations. For rural settings, none of the maternal employment variables were significant. Concerning father’s occupation, there was an excess mortality risks amongst infants whose fathers and mothers were not employed (HR = 2.50, CI: 0.67–9.29), compared to those with professional mothers and unemployed fathers. Infants whose fathers were employed in professional and services and mothers engaged in agriculture and manual work had higher risks of infant death (HR = 1.83, CI: 1.01–3.29). Interaction between maternal employment variables and region are summarised in Table 6b. Infants from women who were not working or employed in agriculture and manual labour had higher risk of death in the north-central region (HR = 1.32, CI: 0.72–2.42), the opposite pattern was observed in the south-east (HR = 0.45, CI: 0.28–0.71). Results further showed that the risk of infant death was higher amongst women not working or earning cash in the north-central (HR = 1.47, CI: 1.09–1.98) and the south-east (HR = 1.89, CI: 1.42–2.52) regions. In the south-south and south-west regions, infants whose fathers were employed in agriculture and manual labour had higher risk of death.

**Table 6a:** Interaction effects of maternal occupation, residence and father’s occupation on infant mortality in Nigeria.

Variables	Residence				Father’s Occupation					

	Urban		Rural		Not Working	Professional/Services		Sales	Agriculture/Manual	

	HR (95% CI)		HR (95% CI)		HR (95% CI)	HR (95% CI)		HR (95% CI)	HR (95% CI)	

**Maternal Occupation**										
not working	0.63 (0.36–1.11)		0.87 (0.59–1.29)		2.50 (0.67–9.29)	1.14 (0.55–2.39)		0.71 (0.27–1.81)	0.65 (0.43–0.97)	*
professional/services	1.00		1.00		1.00	1.00		1.00	1.00	
sales	1.13 (0.78–1.64)		0.79 (0.54–1.16)		1.40 (0.51–3.88)	1.62 (0.95–2.76)		0.87 (0.53–1.43)	0.71 (0.49–1.03)	
agriculture/manual	1.03 (0.71–1.51)		0.86 (0.57–1.28)		1.70 (0.52–5.56)	1.83 (1.01–3.29)	*	0.96 (0.61–1.49)	0.69 (0.48–0.99)	*
**Involvement in Decision on Earnings**										
involved	1.00		1.00		1.00	1.00		1.00	1.00	
not involved	1.24 (0.89–1.73)		0.78 (0.53–1.13)		0.99 (0.14–7.08)	0.84 (0.47–1.49)		0.81 (0.43–1.51)	0.90 (0.68–1.21)	
not working/not earning cash	1.94 (1.34–2.80)		0.89 (0.74–1.07)		0.45 (0.17–1.15)	1.61 (0.89–2.89)		0.99 (0.41–2.36)	1.15 (0.91–1.45)	
**Father’s Occupation**										
not working	0.52 (0.29–0.94)	*	1.08 (0.71–1.64)							
professional/services	1.00		1.00							
sales	0.79 (0.53–1.18)		1.21 (1.01–1.45)	*						
agriculture/manual	1.14 (0.87–1.50)		1.15 (0.99–1.35)							

* p < 0.05.

**Table 6b:** Interaction effects of maternal occupation and geo-political region on infant mortality in Nigeria.

Variables	North-Central		North-East		North-West		South-East		South-South	South-West	

	HR (95% CI)		HR (95% CI)		HR (95% CI)		HR (95% CI)		HR (95% CI)	HR (95% CI)	

**Maternal Occupation**											
not working	1.32 (0.72–2.42)		0.98 (0.65–1.48)		1.63 (0.89–2.98)		0.45 (0.28–0.71)	*	1.08 (0.28–4.21)	1.01 (0.32–3.13)	
professional/services	1.00		1.00		1.00		1.00		1.00	1.00	
sales	1.12 (0.65–1.95)		1.09 (0.64–1.86)		0.88 (0.46–1.67)		1.01 (0.62–1.66)		1.27 (0.57–2.83)	0.68 (0.42–1.09)	
agriculture/manual	1.27 (0.75–2.15)		1.15 (0.82–1.59)		0.89 (0.47–1.69)		0.97 (0.53–1.78)		1.09 (0.64–1.86)	0.72 (0.37–1.39)	
**Mother’s Involvement in Decision on Earnings**											
involved	1.00		1.00		1.00		1.00		1.00	1.00	
not involved	1.06 (0.64–1.77)		0.98 (0.59–1.61)		0.73 (0.39–1.33)		0.90 (0.51–1.86)		1.25 (0.61–2.56)	0.76 (0.33–1.75)	
not working/not earning cash	1.47 (1.09–1.98)	*	0.73 (0.60–0.87)	*	0.56 (0.39–0.82)	*	1.89 (1.42–2.52)	*	1.40 (0.67–2.92)	0.85 (0.28–2.55)	
**Father’s Occupation**											
not working	0.52 (0.18–1.48)		0.69 (0.13–3.70)		0.97 (0.49–1.92)		0.97 (0.51–1.86)		1.29 (0.74–2.25)	0.86 (0.34–2.18)	
professional/services	1.00		1.00		1.00		1.00		1.00	1.00	
sales	1.16 (0.71–1.92)		1.27 (0.83–1.93)		0.96 (0.65–1.41)		0.76 (0.47–1.25)		0.87 (0.47–1.63)	1.07 (0.47–2.43)	
agriculture/manual	1.12 (0.74–1.71)		1.03 (0.74–1.44)		0.95 (0.72–1.24)		1.19 (0.96–1.67)		1.54 (0.74–3.21)	1.51 (1.18–1.93)	*

* p < 0.05.

#### Child Mortality (12–59 Months)

In urban areas, child mortality risk was greatest in children whose mothers were not working (HR = 3.34, CI: 1.33–8.41), but for rural areas, children of saleswomen had the highest risk (Table 7a). With professional fathers, child mortality was significantly more likely when the mother’s occupation was either sales (HR = 1.95, CI: 1.11–3.41) or agriculture and manual (HR = 3.49, CI: 1.31–9.33).

**Table 7a:** Interaction effects of maternal occupation, residence and father’s occupation on infant mortality in Nigeria.

Variables	Residence				Father’s Occupation				

	Urban		Rural		Not Working	Professional/Services		Sales	Agriculture/Manual

	HR (95% CI)		HR (95% CI)		HR (95% CI)	HR (95% CI)		HR (95% CI)	HR (95% CI)

**Maternal occupation**									
not working	3.34 (1.33–8.41)	*	1.29 (0.59–2.84)		SS	1.87 (0.42–8.27)		3.99 (0.73–21.69)	1.13 (0.46–2.78)
professional/services	1.00		1.00		1.00	1.00		1.00	1.00
sales	1.85 (1.01–3.41)	*	1.95 (1.11–3.41)	*	0.15 (0.01–2.64)	3.89 (1.57–9.67)	*	1.81 (0.60–5.39)	1.72 (0.95–3.11)
agriculture/manual	0.96 (0.39–2.33)		1.77 (0.98–3.19)		0.15 (0.01–2.81)	3.49 (1.31–9.33)	*	1.16 (0.38–3.57)	1.49 (0.78–2.83)
**Involvement in Decision on Earnings**											
involved	1.00		1.00		1.00	1.00		1.00	1.00
not involved	1.36 (0.72–2.58)		0.83 (0.56–1.22)		SS	1.05 (0.48–2.33)		1.04 (0.43–2.47)	0.84 (0.59–1.18)
not working/not earning cash	0.87 (0.59–1.29)		0.84 (0.58–1.22)		3.66 (0.32–42.49)	1.37 (0.56–3.34)		0.68 (0.34–1.35)	0.79 (0.54–1.16)
**Father’s Occupation**											
not working	0.32 (0.09–1.20)		0.58 (0.29–1.16)						
professional/services	1.00		1.00						
sales	0.54 (0.29–0.98)	*	0.91 (0.66–1.24)						
agriculture/manual	0.85 (0.48–1.49)		0.79 (0.63–1.01)						

SS = small sample size; * p < 0.05.

Interaction effects between maternal employment and region are presented in Table 7b. It shows that in the north-west region, sales (HR = 3.31, CI: 1.19–9.18) and agriculture/manual occupation (HR = 2.68, CI: 0.90–8.01) were risk factors for child mortality. None of the interaction effects in the north-east, south-east, and south-south regions were statistically relevant.

**Table 7b:** Interaction effects of maternal occupation and geo-political region on child mortality in Nigeria.

Variables	North-Central		North-East	North-West		South-East	South-South	South-West	

	HR (95% CI)		HR (95% CI)	HR (95% CI)		HR (95% CI)	HR (95% CI)	HR (95% CI)	

**Maternal Occupation**									
not working	SS		1.01 (0.47–2.14)	1.56 (0.59–4.14)		0.61 (0.25–1.51)	1.98 (0.63–6.23)	SS	
professional/services	SS		1.00	1.00		1.00	1.00	1.00	
Sales	SS		1.23 (0.59–2.55)	3.31 (1.19–9.18)	*	1.20 (0.75–1.91)	0.99 (0.27–3.58)	2.15 (0.57–8.07)	
agriculture/manual	SS		0.94 (0.40–2.20)	2.68 (0.90–8.01)		0.82 (0.48–1.42)	1.29 (0.33–5.09)	1.00 (0.19–5.28)	
**Involvement in Decision on Earnings**									
involved	1.00		1.00	1.00		1.00	1.00	1.00	
not involved	0.69 (0.51–0.94)	*	1.28 (0.68–2.39)	0.74 (0.26–2.09)		0.91 (0.41–2.01)	1.93 (0.80–4.63)	0.89 (0.27–3.01)	
not working/not earning cash	0.25 (0.07–0.94)	*	0.69 (0.41–1.18)	1.58 (0.92–2.71)		1.13 (0.70–1.81)	1.16 (0.56–2.44)	0.32 (0.01–15.55)	
**Father’s Occupation**									
not working	SS		0.70 (0.13–3.64)	0.81 (0.36–1.78)		0.35 (0.10–1.21)	0.91 (0.15–5.71)	0.60 (0.13–2.87)	
professional/services	1.00		1.00	1.00		1.00	1.00	1.00	
sales	0.49 (0.07–3.63)		1.31 (0.84–2.04)	0.87 (0.61–1.23)		0.55 (0.19–1.58)	0.65 (0.15–2.72)	0.04 (0.01–0.36)	*
agriculture/manual	0.92 (0.43–1.95)		1.12 (0.73–1.73)	0.78 (0.60–1.02)		0.60 (0.34–1.07)	0.87 (0.32–2.35)	0.71 (0.28–1.89)	

SS = small sample size; *p < 0.05.

## Discussion

Previous studies on the relationship between maternal employment and child health outcome have yielded mixed results. This study therefore contributes new evidence on the link between the two. The results showed that infant mortality was higher amongst children of unemployed women, and the difference was greater during 12–59 months of life. There was no significant difference in the infant mortality risk across different maternal occupational groups. Risk of child death (12–59 months) was higher amongst children whose mothers’ occupation was sales or agriculture and manual labour. Father’s occupation was not independently associated with children survival.

A negative effect of maternal unemployment on child mortality is consistent with previous studies on child health outcomes such as nutritional status [17]. The weaker relationship in the first 11 months of life also agree with prior evidence which suggested that socio-economic variables exert greater influence on child survival from one year onward [36]. Post infancy, children need complementary foods choices which may not be easily afforded by unemployed women. This could result in undernutrition, which is responsible for 45% of childhood deaths in developing countries [37].

Greater risks of child mortality amongst women engaged in sales, agriculture, and manual work could be a result of inadequate attention to childcare. These occupational groups have also been found to have the poorest coverage of childhood vaccination [27]. Though these are mostly informal occupations that should allow mothers to make time for children’s care, the results of this study suggest otherwise.

Findings from this study are a sharp contrast to previous evidence [9,10] that indicated maternal employment was a risk for infant mortality. The difference could be attributed to two reasons. First, there has been some improvement in childcare practices and health interventions in the past two decades, such that a negative effect of maternal employment could have been reversed [38]. Second, most employed mothers in the previous studies were engaged in the informal sector (agriculture, sales, etc.), which has been further confirmed by this study to be risky for children survival. Sequel to improved female education and several women empowerment activities, more women now get jobs in the formal sector, and this brings some advantages to the family and children [4].

Our results revealed that children of unemployed couples had the greatest mortality risks. Such partners would not be able to provide basic needs such as good diet and prompt treatment of common childhood illnesses. The consequence of their poor economic resources may result in increased child deaths. As a matter of fact, the leading causes of childhood deaths such as malaria have been associated with poverty [39]. Furthermore, the negative effect of maternal unemployment was stronger in urban than rural areas despite the general knowledge that childhood mortality rates are higher in the latter. This may be because of higher cost of living in the urban areas. Previous studies have actually shown that childhood morbidity and mortality could be worse amongst poor urban dwellers than those in rural areas [40,41].

The results further showed that the childhood mortality risk was higher amongst women involved in sales in both rural and urban areas. The same pattern was replicated across all geopolitical regions except the south-east. It is suspected that the modes of operation of sales occupation by women in this region is more compatible and favourable to child survival than it is in the other four regions (north-west, north-east, north-central, and south-west). This may constitute an interesting research question for the future.

A few implications of the findings are obvious, especially for SDGs 3 and 5. Of research importance is the need to uncover reasons why childhood mortality was higher amongst women in occupations such as sales, agriculture, and manual labour. Childcare coping strategies and practices amongst these women would need to be better understood. This is necessary so that appropriate interventions can be designed to reach this group of women.

About policy and programmes, women unemployment needs to be addressed as a development priority. This study has demonstrated synergy between SDG 3 and 5. Increased participation of women in the formal labour force will positively affect child survival. Because the majority of women are employed in the informal sector (sales, agriculture, and manual work), it may be beneficial to design special advocacy programmes on child survival and targeted at these group of women. They often have organised groups and associations which may be partnered for outreach on children healthcare and survival. This is very relevant in view of recent economic challenges and the attendant pleas for Nigerians to embrace agriculture.

A few limitations of this study are noteworthy. Childhood mortality differentials according to occupation may be related to variation in child care practices. However, there was no data to control for these during analysis. Further, we did not embark on a deep exploration of the pathways through which maternal employment affect child survival. It is suspected that child healthcare utilisation has roles to play. Aside these limitations, this study has successfully renewed the necessary attention towards a neglected but important subject matter.

On a final note, this study demonstrated variations by residence and region in the effect of maternal unemployment on childhood mortality. Also, though father’s occupation did not show any independent relationship with infant and child mortality, children of unemployed parents have the greatest risks of death during infancy. Last, sales and agriculture or manual work constitute threats to children survival. Because labour laws for the formal sector and occupational practices in the informal sector may vary in other countries, it may be useful to conduct similar studies in other sub-Saharan Africa countries. Such contextual evidence is necessary for culturally sensitive child survival interventions and programmes. Increased participation of women in formal occupation holds a favourable outlook for child survival in Nigeria.

## Author Information

All authors had access to the data and played a role in writing the manuscript.
